# Plant Milking Technology—An Innovative and Sustainable Process to Produce Highly Active Extracts from Plant Roots

**DOI:** 10.3390/molecules25184162

**Published:** 2020-09-11

**Authors:** Hanane Chajra, Aleksander Salwinski, Agnès Guillaumin, Benoit Mignard, Paul Hannewald, Léonor Duriot, Pierre Warnault, Carine Guillet-Claude, Mathilde Fréchet, Frédéric Bourgaud

**Affiliations:** 1 Clariant Active Ingredients, 195 Route d′Espagne, 31000 Toulouse, France; benoit.mignard@clariant.com (B.M.); mathilde.frechet@clariant.com (M.F.); 2 Plant Advanced Technologies, 19 Avenue de la Forêt de Haye, 54500 Vandoeuvre, France; aleksander.salwinski@plantadvanced.com (A.S.); agnes.guillaumin@plantadvanced.com (A.G.); paul.hannewald@plantadvanced.com (P.H.); leonor.duriot@plantadvanced.com (L.D.); pierre.warnault@plantadvanced.com (P.W.); Carine.Guillet-Claude@plantadvanced.com (C.G.-C.); frederic.bourgaud@plantadvanced.com (F.B.)

**Keywords:** natural compound, root, sustainability, moracenin, *Morus alba* L., prenylated flavonoids

## Abstract

We have used an original technology (Plant Milking Technology) based on aeroponic cultivation of plants associated with the gentle recovery of active ingredients from roots. Extraction of bioactive molecules was achieved by soaking the roots, still attached to the living plants, into a nontoxic solvent for a 2 h period. This nondestructive recovery process allows using the same root biomass for successive harvesting dates, in a recyclable way. We have applied this technology to *Morus alba* L. (mulberry tree), an emblematic tree of the Traditional Chinese Medicine (TCM). Trees were aeroponically grown in large-scale devices (100 m^2^) and were submitted to nitrogen deprivation to increase the content in active molecules (prenylated flavonoids). The Plant Milking technology applied to *Morus alba* L. allowed to produce an extract enriched in prenylated compounds (18-fold increase when compared to commercial root extract). Prenylated flavonoids (moracenin A and B, kuwanon C, wittiorumin F, morusin) presented a high affinity for the aged-associated collagenase enzyme, which was confirmed by activity inhibition. In accordance, *M. alba* extract presents efficient properties to regulate the skin matrisome, which is critical during skin aging. The benefits have been especially confirmed in vivo on wrinkle reduction, in a clinical study that involved aged women. Plant Milking technology is an optimal solution to produce active ingredients from plant roots, including trees, that meet both customer expectations around sustainability, as well as the need for an efficient production system for biotechnologists.

## 1. Introduction

Roots from terrestrial plants constitute a metabolically active yet difficult to exploit compartment given their underground characteristic. The high importance of root phytochemical diversity is clear from the broad use of this plant compartment in traditional medicine across the globe [1,2]. Root apices constitute the main parts where active substances are produced and stored in roots [3]. They are almost impossible to harvest through the use of conventional farming methods. Moreover, alternative sourcing through the chemical synthesis of structurally complexed molecules is not always economically feasible, due to the multiple reaction steps required to yield the final product. Biotechnological advances have given us the opportunity to use the cells, tissues and cultures of economically important species and to genetically manipulate them in order to obtain the desired compounds [4]. However, serious concerns about biotechnologies are sometimes expressed by customers who have a high demand for “wise technologies.” This dynamic comes as a result of environmental awareness around the preservation of biodiversity and ecosystems, the steps being taken to achieve a reduced carbon footprint and the use of non-GMO plants and so forth.

The purpose of this study was to (i) assess the possibility of producing a root extract in an aeroponic cultivation system implemented at large scale (100 m^2^); (ii) compare both qualitatively and quantitatively the phytochemical profile of this extract to the one obtained from a sample of the same plant grown in soil; (iii) evaluate the effect of this extract on skin matrisome through the study on collagenase enzyme and expression of genes related to matrisome; and (iv) further confirm the biological activity of the extract by clinical trials on human volunteers.

The aeroponic cultivation device used in this study allows a prolific growth of plant roots and offers easy access to the root compartment. While still attached to the plants, roots are gently soaked into a compatible solvent for a brief period, usually around 2 h, the exact duration depending on each plant species and the type of biomolecule to be recovered. This process is a non-destructive harvest for the plants, ensuring that they can be used indefinitely to recover the desired molecules of interest. This so-called “Plant Milking technology” has been applied to *Morus alba* L., a tree emblematic of the Traditional Chinese Medicine (TCM). The root extract obtained from aeroponically-grown plants was highly enriched in prenylated molecules (18-fold increased concentration) when compared to already available commercial root extract. This extract was proven to be highly biologically active and can be proposed as an effective cosmetic active ingredient with anti-aging properties for the skin.

## 2. Results

### 2.1. Plant Cultivation Performance

**Figure 1:**
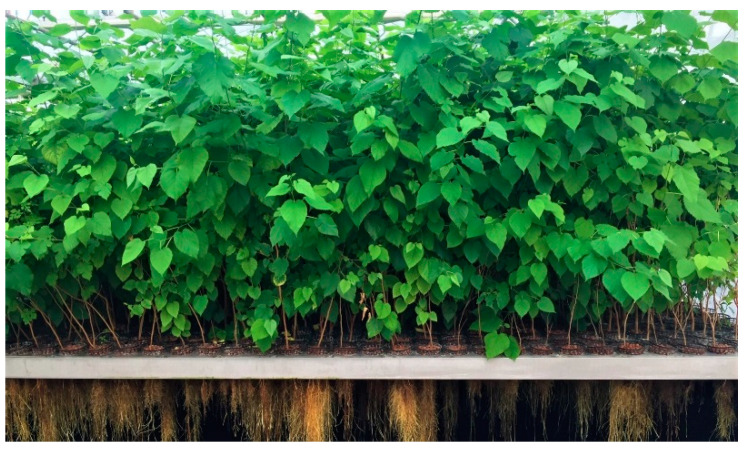
*Morus alba* L. trees cultivated in aeroponic conditions.

**Figure 2:**
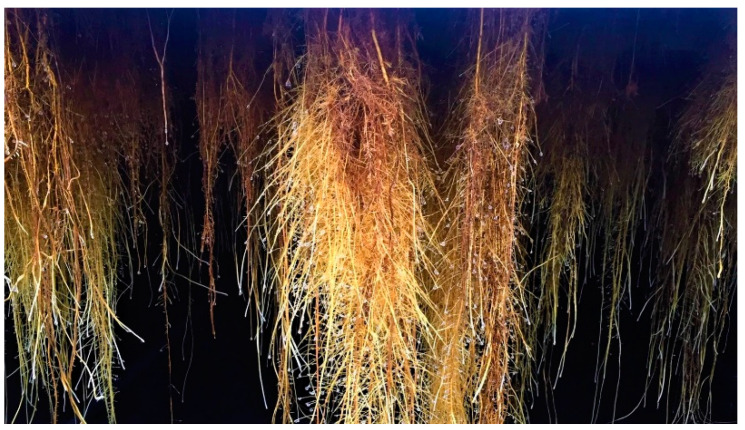
Close-up view of *Morus. alba* L. roots grown aeroponically.

Three-year old *M. alba* trees cultivated in aeroponic conditions produced a prolific biomass for both roots and aerial parts (Figure 1). Thin roots were abundant and presented a pronounced yellow color (Figure 2).

Nitrogen deficiency was previously described to enhance the synthesis of polyphenolic compounds in plants like Arabidopsis [5] and Tomato [6]. Based on these findings, it was decided to apply temporary nitrogen deprivation to *M. alba* trees grown aeroponically with the objective to increase the content of moracenin and structurally related molecules which have a polyphenolic skeleton. No visual differences in root biomass production could be observed between control plants (normal nitrogen nutrition) and plants submitted to nitrogen limitation for 4 weeks.

### 2.2. Plant Metabolite Quantification and Comparison with Commercial Sample

**Figure 3:**
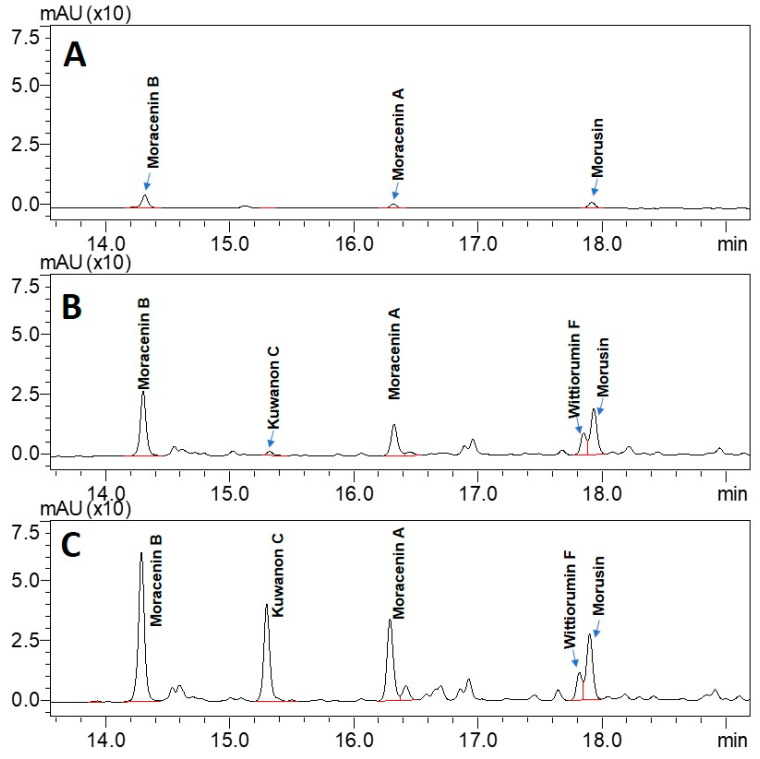
UHPLC chromatograms of the extracts obtained from commercially available roots (**A**), from control roots cultivated in aeroponic system (**B**) and from roots stimulated with nitrogen deficiency (**C**). All chromatograms were acquired at 265 nm.

Ultra-high performance liquid chromatography (UHPLC) analyses revealed the occurrence of five major compounds in *M. alba* root extract (Figure 3B). These molecules were identified using negative ion mode mass spectrometry based on the detection of pseudo-molecular ions [M − H]^−^. Molecular structures were confirmed by nuclear magnetic resonance (NMR) analyses (see Appendix B for NMR determination). These five molecules were unambiguously determined as moracenin B, kuwanon C, moracenin A, wittiorumin F and morusin. The chemical structure of each molecule is presented in Figure A9. The concentrations of these 5 molecules in plant root extracts were determined on the basis of the peak areas at 265 nm (Figure 3) using a standard solution of moracenin B at 100 mg/L as a reference. The concentrations are thus provided as equivalent of moracenin B.

**Figure 4:**
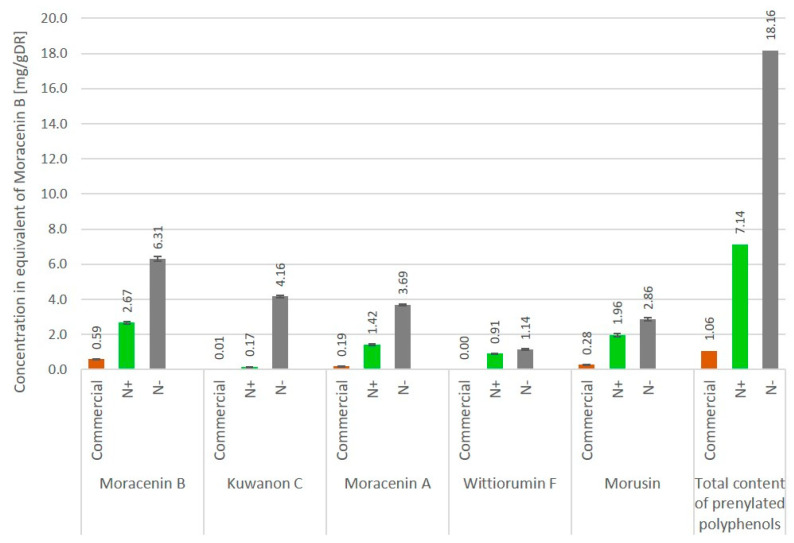
Concentration of metabolites in *Morus. alba* L. roots cultivated under aeroponic soil-less conditions with and without nitrogen deficiency (N-/N+ respectively) and commercially available roots. Concentrations are expressed as the quantity of individual markers per 1 g of dry root powder, mg/gDR (*n* = 3).

The overall content of five *M. alba* markers (moracenin B, kuwanon C, moracenin A, wittiorumin F and morusin) in the roots of the aeroponically cultivated plants was 7-times higher for non-nitrogen depleted plants and 18-times higher for nitrogen deprived plants when compared to commercial mulberry roots (Figure 4).

### 2.3. Plant Metabolites Reveal an Affinity for the Collagenase

**Figure 5:**
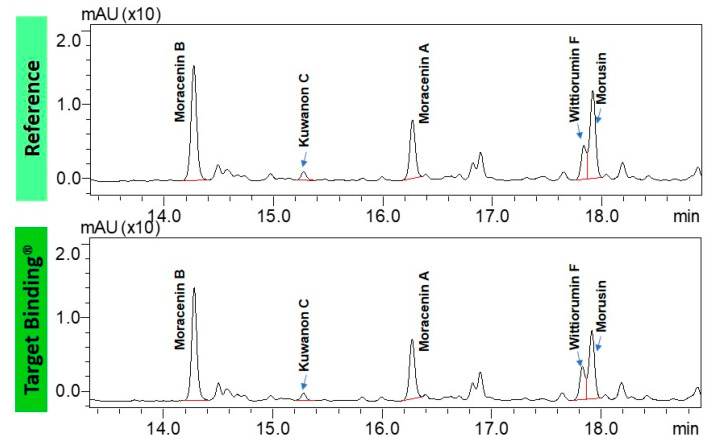
UHPLC chromatograms of the test mixture: *Morus alba* L. root extract cultivated in aeroponic system (**top**) and the supernatant containing the compounds binding to collagenase from *Clostridium histolyticum* (**bottom**).

As collagenase is an enzyme involved in skin deterioration along with aging process, we tried to determine if the phytochemical compounds found in root extracts from nitrogen depleted plants (N-) have the ability to inhibit collagenase activity by physically interacting with this enzyme. Target binding using the collagenase allows for the identification of the phytochemical compounds that interact with this enzyme. The comparison of the chromatograms of the test mixture and the supernatant containing the compounds retained by collagenase, shown in Figure 5, leads to the conclusion that all major prenylated polyphenols in the extract bind the enzyme. Moreover, we determined relative affinity (RA) for collagenase for each prenylated compound (Table 1). The RA value for the reference compound, represented here by moracenin B, always equals one. The RA values are proportional to the affinity of the compounds to the target. The compounds containing RA values that are higher than 1.0, show a higher affinity than the reference compound. The affinities of *Morus alba* L. metabolites to collagenase are very close to the affinity of moracenin B used as the benchmark and oscillate at around a value of 1.0. Morusin and kuwanon C show slightly lower affinity than moracenin B, while the affinity of moracenin A, wittiorumin F is slightly higher.

### 2.4. The Unique Composition of N- Extract Exerts a Synergetic Collagenase Inhibition Effect

After having demonstrated that prenylated compounds found in N-sample have an affinity for collagenase, we checked whether this affinity for collagenase is associated with a functional biological effect, such as the inhibition of collagenase activity. In order to assess this, we first determined the concentration of each prenylated polyphenol that was previously identified in N-. Then, we calculated the percentage of relative collagenase activity (AcTR %). Interestingly, we observed that N- extract has a higher collagenase inhibitory effect (−73%) in comparison to each prenylated molecule tested at the same concentration (Moracenin B: −42%, Kuwanone C: −38%, Morusin: −32% and Moracenin A: −62%). Our results suggest a synergistic effect of these prenylated compounds contained in N- (Table 2). Then, we compared the collagenase inhibitory effect of N-, in comparison to the traditional root extract. This was in order to determine whether this inhibitory effect is provided by the unique N- composition. Traditional root extract cultivated in soil has demonstrated no inhibitory effect in terms of collagenase activity.

### 2.5. N- Extract Regulates the Expression of Key Matrisome Related Genes: CCN1, MMP-1 and COL3A1

N- inhibits the mRNA transcription of cysteine rich angiogenic protein 61 (CCN1) and matrix metalloproteinase 1 (MMP-1) (Table 3). In contrast, N- stimulates mRNA transcription level of collagen 3 alpha 1 subunit (COL3A1).

### 2.6. N- Extract Protects Human Fibroblasts against UV-B-Induced Decline Procollagen Type I C Propeptide (CICP) Production

**Figure 6:**
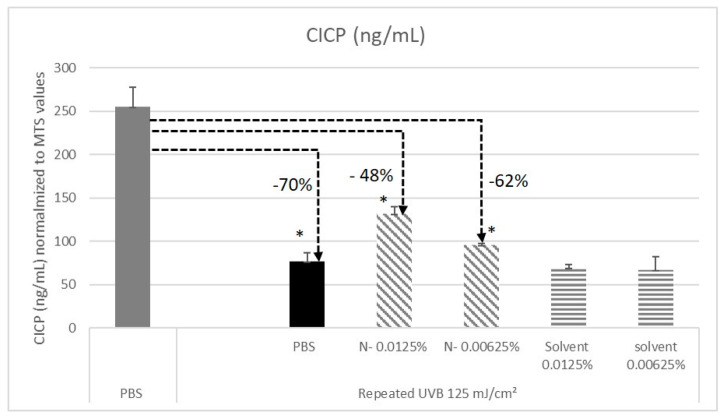
Quantification of Procollagen Type I C Propeptide (CICP) secreted in the supernatant of normal human fibroblasts dermal fibroblasts NHDFs under various conditions. Elisa assay. The study was done in triplicate (* *p* < 0.05, student *t* test).

We addressed the effect of N- on CICP production after exposing fibroblasts to UV-B irradiation, which is a major environmental skin aggressor that is involved in premature aging. Before each UV-B irradiation, the fibroblasts were pretreated with N-. The CICP quantification was performed in the cell′s supernatants. As expected, CICP concentration measured in the UV-B stressed fibroblasts was dramatically reduced by −70% (Figure 6). We observed that when fibroblasts were pretreated by N- root extract, this negative effect was reduced. The benefit provided is dose dependent. These results suggest that even though they are highly impacted by the repeated UV-B irradiation, the capacity of the cells to produce CICP in the supernatant remains functional. This, therefore, demonstrates a protective effect of N- regarding to fibroblasts functionality under UV-B stress.

### 2.7. N- Extract Reduces Wrinkles Depth, Improves Skin Smoothness and Skin Plumping

**Figure 7:**
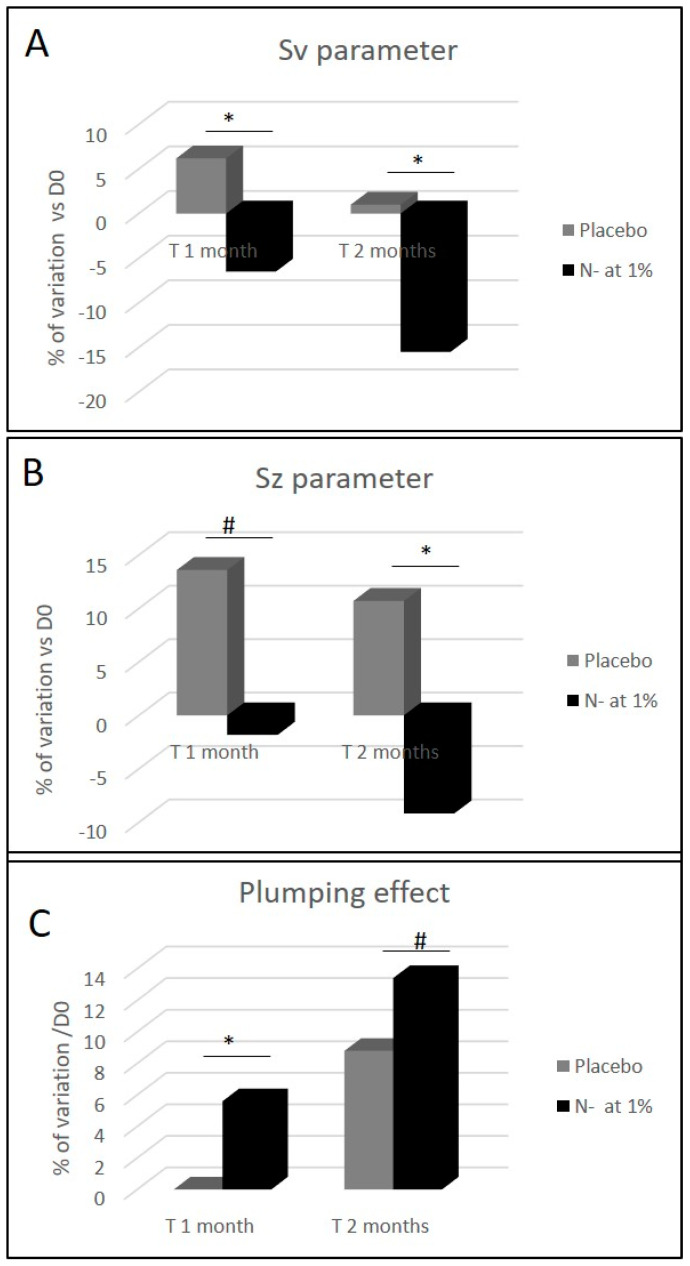
Clinical measurement of skin wrinkles depth (Sv parameter), skin smoothness (Sz parameter) and skin plumping effect before and after 1 and 2 months of a cosmetic cream application containing either placebo or N- root extract (1%) on the face. Sv and Sz parameters determined with C-cube and skin plumping effect, were reported through the use of a grading scale. The scale range varies from 0 (sagging, skin not firm) to 10 (tonic, firm and plumped skin). The measurements were done on the crow’s feet area. (* *p* < 0.05, and ^#^ *p* < 0.1).

Wrinkled skin appearance and rough textured skin are common criteria of photoaged skins [7], which is easily visible in the crow’s feet area. This is why we focused our investigation on N- extract clinical efficacy on its capability to counter the appearance of these type of wrinkles. The volunteers were asked to twice daily apply the cosmetic product at 1% and the placebo cream on their faces for 2 months. After 1 and 2 months of N- root extract use, the analysis of crow’s feet wrinkles with C-Cube showed a statistically significant improvement of skin relief parameters Sv (which represents the deepest wrinkles) and Sz (which represents skin smoothness). After 1 and 2 months, the N- Sv parameter was reduced by −12.7% and −16.6% (Figure 7A) in comparison to placebo group, while the Sz parameter was reduced by −15.4% and−19.9% (Figure 7B). We also observed a time dependent improvement effect for these two parameters for the N- group, in comparison to the placebo group, where an increase was observed. The skin plumping determined by a 10-grade scoring scale was significantly improved, when compared to the placebo (Figure 7C). In addition, we noted that the observed plumping effect is time dependent. Indeed, when considering N- formula, the skin plumping effect was significantly higher after 1 month and even greater after 2 months, when compared to D0 (+5.6% and +13.4% respectively).

## 3. Discussion

Aeroponic plant cultivation systems are soilless devices in which the soil has been replaced by a liquid culture medium that is regularly sprayed onto the roots through the application of a nutritive mist [8]. Our aeroponic cultivation method appears to have an impact on the chemical composition of *Morus alba* L. root extracts, since wittiorumin F completely was missing in the commercial sample obtained from roots of field-grown trees (Figure 3). Another motivation in the use of an aeroponic technique to produce plant natural molecules is to modify the mineral composition of the nutrient medium to boost the biosynthesis of the compounds of interest [9]. In our experiments, nitrogen limitation led to a 7-fold increase of prenylated compound concentration (Figure 4). Compared to a commercial sample of *Morus alba* L, this increase is even more pronounced with a 18-fold enhancement being reported. Another advantage of aeroponic cultivation devices is the immediate access to the root compartment. This therefore turns root harvesting into a non-destructive operation for the trees and allows for the multiple harvesting of the same plants per year of exploitation.

In addition, we demonstrate that this enriched root extract exerts unique anti-aging properties in skin, which is provided in part by the collagenase inhibitory effects of its prenylated compounds. Interestingly, this benefit was not observed in the case of the commercial root extract. To our knowledge, it has never been previously demonstrated that prenylated polyphenols such as moracenin A, moracenin B, wittiorumin F, kuwanon C or morusin exert the ability to bind collagenase or inhibit collagenase activity. In our biological experiments, we studied the effect of N-extract on collagenase, as this enzyme is strongly deregulated during skin intrinsic (genetically programmed chronological aging) [10,11] and extrinsic aging UV rays for example) [12,13].

Human skin is a major target of UV-B assaults [14]. UV-B directly or indirectly generate reactive oxygen species (ROS) in the skin fibroblasts that trigger oxidative stress-mediated skin connective tissue injury, such as a decrease in procollagen synthesis (collagen type I and III for example [15]) and an increase in MMP-1 [16]. This contributes to premature skin aging. Interestingly, it has been demonstrated that this decrease is mediated by CCN-1 [17], a skin matrisome component that is modulated by N- [18,19]. Indeed, it has been demonstrated that the elevated expression of CCN1 in dermal fibroblasts exposed to UV irradiation impairs collagen homeostasis [20]. Researchers also observed that elevated CCN1 rapidly inhibits type I procollagen production and upregulates matrix metalloproteinases (MMP-1, 3 and 9). These are collagenases that are known for their ability to degrade collagen fibrils [17]. Moreover, it has been shown that CCN1 is strongly elevated in sun exposed prematurely aged skin and in aged cells [17].

Furthermore, N- extract also inhibits MMP-1 expression and maintains fibroblasts′ procollagen neosynthesis ability, which is known to be impacted by UV-B rays. The anti-aging properties on the skin were confirmed on human volunteers, with an improvement on the impact of skin wrinkles observed through the topical use of N- root extract.

Our data also demonstrates the clear benefits of protecting skin matrisome components against external aggressions such as UV, pollution or oxidative stress, as a way to limit skin declines that appear with aging. It thus limits wrinkles apparitions, skin thinning, loss of skin elasticity and firmness. Therefore, finding a plant extract such as N- with the ability to mitigate CCN1 expression, may be an efficient solution in the fight against premature aging.

## 4. Materials and Methods

### 4.1. Comparison of Aeroponically Grown Plant Roots with Commercial Sample

Commercial roots of *Morus alba* L. (Cortex Mori Albae Radicis/Sang Bai Pi, CapsulCN International, Ruian, China) were compared to aeroponically grown roots using UHPLC. The extraction process was conducted in the same way for all root samples—commercial roots and roots originating from nitrogen non-deprived (N+) and nitrogen deprived (N-) plants cultivated in aeroponics. Briefly, dry roots were grounded using the ball mill (VWR Beater, 5 min at 20 Hz and 5 min at 30 Hz) and extracted with pure EtOH (20 mg of ground roots/mL) by vortexing at room temperature for 30 min. The samples were then centrifuged at 15,000 rpm/RCF 21,000× *g* for 10 min. Analytical samples were prepared through the 5-fold dilution of the supernatants obtained after centrifugation with pure EtOH. All root samples were extracted and analyzed by UHPLC (Shimadzu, Kyoto, Japan) in triplicate.

### 4.2. Plant Cultivation in Aeroponic Systems

*Morus alba* L. plants were cultivated aeroponically for 8 weeks in a culture medium (Plant-Prod, ref. 209.00) with N/P/K respective ratios of 15/10/30. Then, in the case of nitrogen-deprived plants, *Morus alba* L. trees underwent 3 weeks in a nutrient solution without nitrogen (N/P/K = 0/15/40, supplied by Plant-Prod, ref. 211.00). Both types of plants had their roots dried for 48 h in a ventilated oven at 40 °C.

### 4.3. Solvents

HPLC grade acetonitrile was purchased from Sigma-Aldrich (Steinheim, Germany), reagent grade formic acid, absolute ethanol and technical grade methanol were purchased from Carlo Erba (Val-de-Reuil, France).

### 4.4. Preparation of Crude Extract for Molecule Purification

Dried, ground roots were extracted through the use of absolute ethanol (40 g/L) for 2 h at room temperature by stirring. A volume of 1000 mL of this extract was concentrated under vacuum yielding 3 g of a crude dry extract.

### 4.5. Purification by Preparative Liquid Chromatography

**Figure A1:**
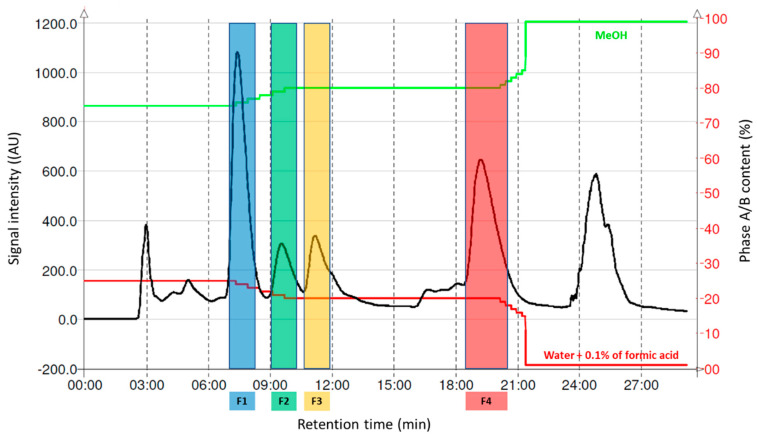
Preparative LC chromatogram of *M. alba* root extract with fractions F1–F4 corresponding to moracenin B (F1), moracenin A (F2), kuwanon C (F3) and morusin (F4).

A mass of 765 mg of the crude extract was then dissolved in 7 mL of absolute ethanol and 1 mL water containing 0.1% of formic acid. The resulting solution was then separated using preparative LC Armen Spot Prep II (Armen, Saint-Avé, France) with a C18 column (250 mm × 50 mm, 10 µm, Vydac Denali, Grace, Columbia, MD, USA), with an UV detection at 268 and 400 nm and a flow rate of 120 mL/min. The crude extract was injected three times. Mobile phase was composed of water containing 0.1% vol. of formic acid (A) and pure methanol (B) with the gradient of B phase as follows: 75% (0–7 min), 75–80% (7–10 min), 80% (10–20 min), 80–99% (20–21 min), 99% (21–25 min). The individual fractions F1–F4, (Appendix A Figure A1), corresponding respectively to prenylated polyphenols: moracenin B(F1), moracenin A (F2), kuwanon C (F3) and morusin (F4) were pooled, evaporated under vacuum and lyophilized. Wittiorumin F was purified with the same parameters except for the flow rate of 80 mL/min and for the gradient of phase B: 75–95% (0–20 min), 95–100% (20–21 min), 100% (21–31 min).

### 4.6. Structural Elucidation of M. alba L. Metabolites (UHPLC-MS and NMR Analyses)

NMR experiments were performed on a Bruker Avance III 400 spectrometer (Bruker, Billerica, MA, USA) equipped with a BBFO probe (Bruker, Billerica, MA, USA) and operating at a 400.13 MHz frequency for proton. Analyses were performed on the Plateforme de RMN de l′Institut Jean Barriol, Université de Lorraine.

The following parameters are used for ^1^H experiments: number of scans 64, number of points: 65,536, spectral width: 6000 Hz, repetition time: 1.5 s. For ^13^C experiments, a power gated decoupling pulse sequence are used with the following parameters: number of scans 8192, number of points: 65,536, spectral width: 24,000 Hz, repetition time: 2 s. HSQC experiments were performed with the following parameters: number of scans: 32, number of points: 1024 for the ^1^H dimension and 256 for the indirect (^13^C) dimension, spectral width: 6000 Hz for ^1^H, 24,000 Hz for ^13^C, repetition time: 1.5 s.

For these analyses, 10 mg of the different samples were dissolved in 600 µL of CD3OD solvent.
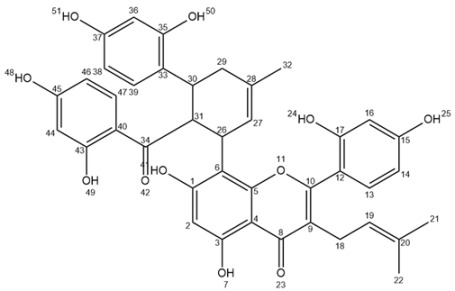


**Figure A2:**
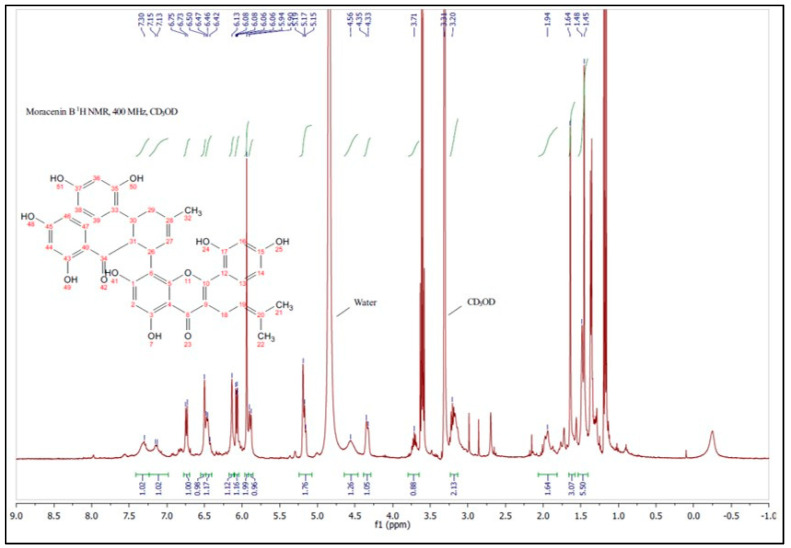
^1^H-NMR (400 MHz, CD_3_OD) spectrum of the compound Moracenin B.

Moracenin B/Kuwanon G/Albanin F (Appendix B Figure A2, F1): 19 mg; yellow powder; purity (UV) 93%; APCI-ESI MS *m/z* (relative intensity) 691 [M − H]^−^ (100); UV λ_max_ 264 and 320 nm; ^1^H-NMR (400 MHz, CD_3_OD) δ 7.30 (s, 1H, H47), 7.14 (*d*, *J* = 8 Hz, 1H, H13), 6.74 (*d*, *J* = 8 Hz, 1H, H39), 6.50 (s, 1H, H16), 6.46 (*d*, *J* = 8 Hz, 1H, H14), 6.13 (s, 1H, H2), 6.07 (*dd*, *J* = 8, 2 Hz, 1H, H38 or H46), 5.94 (s, 2H, H36/H44), 5.89 (*d*, *J* = 8 Hz, 1H, H38 or H46), 5.19 (s, 1H, H19), 5.16 (*d*, *J* = 8 Hz, 1H, H27), 4.56 (s, 1H, H31), 4.34 (*d*, *J* = 8 Hz, 1H, H26), 3.71 (s, 1H, H30), 3.19 (s, 2H, H18), 1.94 (s, 2H, H29), 1.64 (s, 3H), 1.48 (s, 3H, H32), 1.45 (s, 3H).
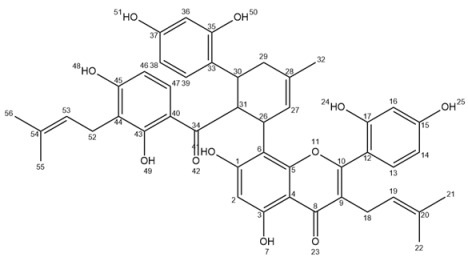


Moracenin A/Kuwanon H/Albanin G (Appendix B, F2): 14 mg; yellow powder, purity (UV) 78%; APCI-ESI MS *m/z* (relative intensity) 759 [M − H]^−^ (100); UV λ_max_ 262 nm; The NMR spectrum (^1^H-NMR, 400 MHz, CD_3_OD) of moracenin A is the same as NMR spectrum than moracenin B except for: the signal at 5.94 ppm integers for only 1 proton, the following signals are additional signals: 5.05 (*t*, *J* = 6.6 Hz, 1H, H53), 3.10 (*d*, *J* = 6.6 Hz, 2H, H52), 1.66 (s, 3H), 1.59 (s, 3H).
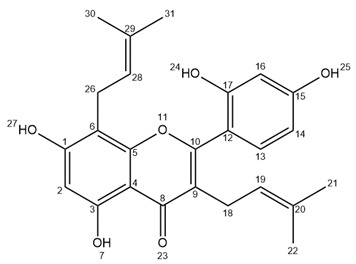


**Figure A4:**
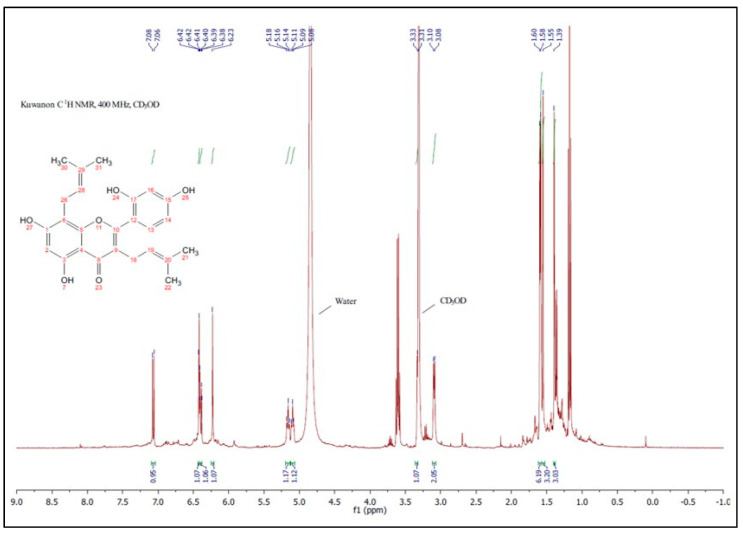
^1^H-NMR (400 MHz, CD_3_OD) spectrum of the compound Kuwanon C.

Kuwanon C/Mulberrin (Appendix B Figure A4, F3): 11 mg; yellow powder; purity (UV) 76%; APCI-ESI MS *m/z* (relative intensity) 421 [M − H]^−^ (100); UV λ_max_ 262 nm; ^1^H-NMR (400 MHz, CD_3_OD) δ 7.07 (*d*, *J* = 8.3 Hz, 1H, H13), 6.42 (*d*, *J* = 2.1 Hz, 1H, H16), 6.39 (*dd*, *J* = 8.3, 2.1 Hz, 1H, H14), 6.23 (s, 1H, H2), 5.16 (*t*, *J* = 7.3 Hz, 1H, H28), 5.10 (*t*, *J* = 6.8 Hz, 1H, H19), 3.32 (*d*, *J* = 7.3, 2H, H26), 3.09 (*d*, *J* = 6.8 Hz, 2H, H18), 1.60 (s, 3H), 1.58 (s, 3H), 1.55 (s, 3H), 1.39 (s, 3H).
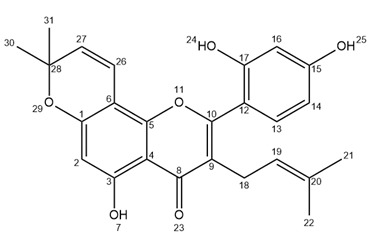


**Figure A5:**
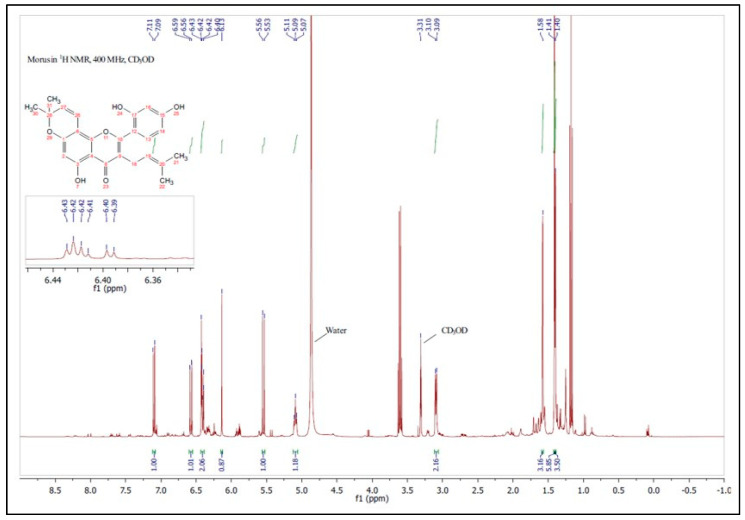
^1^H-NMR (400 MHz, CD_3_OD) spectrum of the compound Morusin.

**Figure A6:**
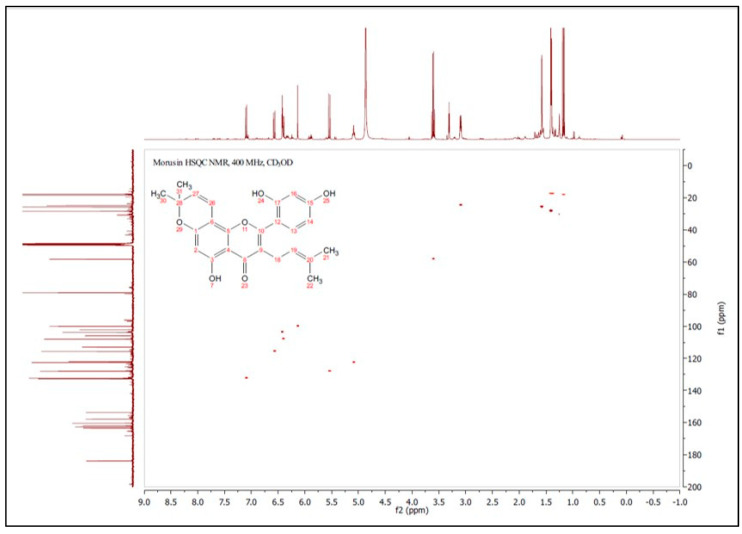
H-SQC NMR (CD_3_OD) spectrum of the compound Morusin.

**Figure A7:**
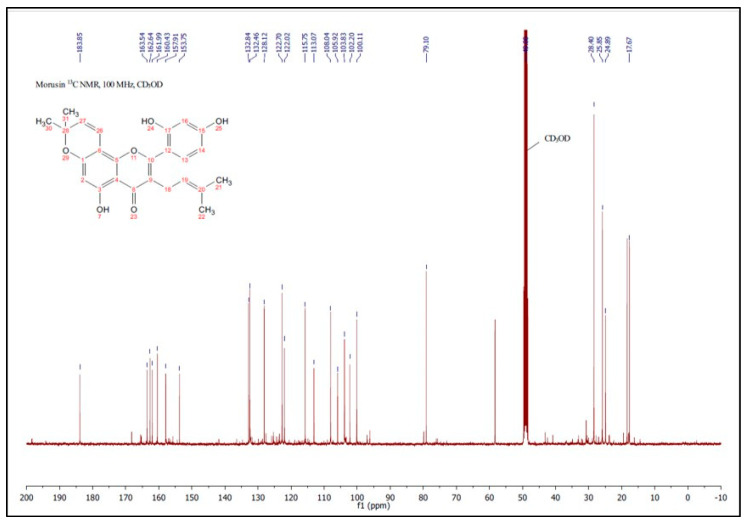
^13^C-NMR (100 MHz, CD_3_OD) spectrum of the compound Morusin.

Morusin (Appendix B Figure A5, Figure A6 and Figure A7, F4): 26 mg; yellow powder; purity (UV) 86%; APCI-ESI MS *m/z* (relative intensity) 419 [M − H]^−^ (100), 839 [2M − H]^−^ (10); UV λ_max_ 268 nm; ^1^H-NMR (400 MHz, CD_3_OD) δ 7.10 (*d*, *J* = 8.4 Hz, 1H, H13), 6.57 (*d*, *J* = 10.0 Hz, 1H, H26), 6.42 (*d*, *J* = 2.4 Hz, 1H, H16), 6.41 (*dd*, *J* = 8.4, 2.4 Hz, 1H, H14), 6.13 (s, 1H, H2), 5.54 (*d*, *J* = 10.0 Hz, 1H, H27), 5.09 (*t*, *J* = 7.0 Hz, 1H, H19), 3.09 (*d*, *J* = 7.0 Hz, 2H, H18), 1.58 (s, 3H), 1.41 (s, 6H, H30/H31), 1.40 (s, 3H).; ^13^C-NMR (100 MHz, CD_3_OD) δ ppm 183.85, 163.54, 162.64, 161.99, 160.43, 157.91, 153.75, 132.84, 132.46 (C13), 128.12 (C27), 122.70 (C19), 122.02, 115.75 (C26), 113.07, 108.04 (C14), 105.92, 103.83 (C16), 102.20, 100.11 (C2), 79.10, 28.40 (2C, C30/C31), 25.85 (C21), 24.89 (C18), 17.67 (C22).
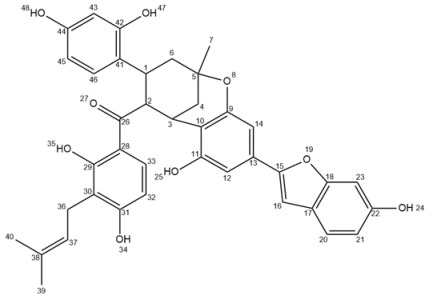


**Figure A8:**
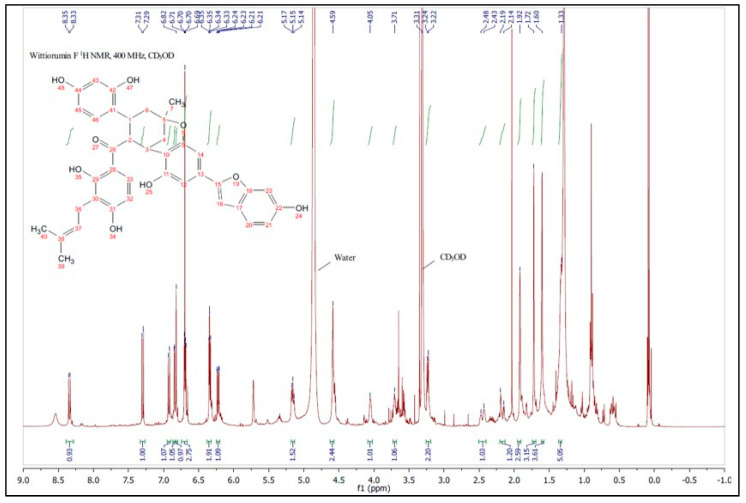
^1^H-NMR (400 MHz, CD_3_OD) spectrum of the compound Wittiorumin F.

**Figure A9:**
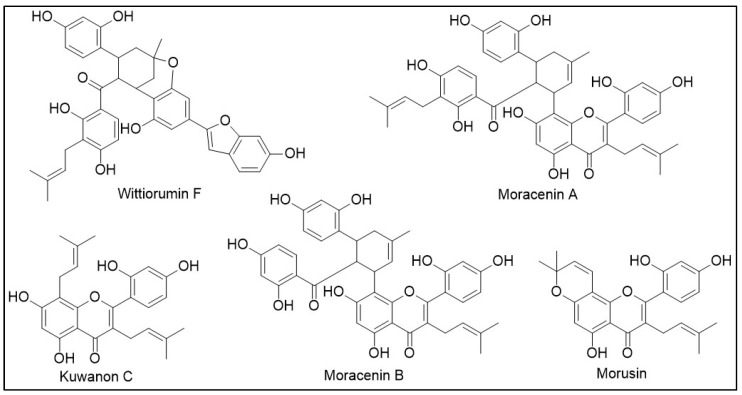
Chemical structures of the molecules moracenin B, kuwanon C, moracenin A, wittiorumin F and morusin.

Wittiorumin F (Appendix B Figure A8): purity (UV) 85%; APCI-ESI MS *m/z* (relative intensity) 647 [M − H]^−^ (100); UV λ_max_ 320 and 220 nm; ^1^H-NMR (400 MHz, CD_3_OD) δ 8.34 (*d*, *J* = 9.2 Hz, 1H, H33), 7.30 (*d*, *J* = 8.4 Hz, 1H, H20), 6.92 (*d*, *J* = 8.4 Hz, 1H, H46), 6.84 (*d*, *J* = 2.1 Hz, 1H, H23), 6.82 (s, 1H, H16), 6.70 (s, 2H, H12/H14), 6.69 (*dd*, *J* = 8.4, 2.1 Hz, 1H, H21), 6.34 (*d*, *J* = 9.2 Hz, 1H, H32), 6.34 (*d*, *J* = 2.5 Hz, 1H, H43), 6.22 (*dd*, *J* = 8.4, 2.5 Hz, 1H, H45), 5.15 (*t*, *J* = 7.2 Hz, 1H, H37), 4.59 (m, 1H, H2), 4.05 (m, 1H, H3), 3.71 (m, 1H, H1), 3.23 (*d*, *J* = 7.2 Hz, 2H, H36), 2.45 (*d*, *J* = 18.7 Hz, 1H6a), 2.17 (*d*, *J* = 18.7 Hz, 1H6b), 1.92 (s, 2H, H4), 1.72 (s, 3H), 1.60 (s, 3H), 1.33 (s, 3H).

### 4.7. Preparation of the Sample for Biological Evaluation of the Extract

Briefly, dry roots of nitrogen deprived *Morus alba* L. (N-) were grounded using the ball mill (VWR Beater, 5 min at 20Hz and 5 min at 30 Hz) and extracted with the solvent composed of 1,3-propanediol/water 80/20 *v/v* (50 mg of ground roots/mL) by agitation at room temperature for 2 h. The samples were then filtered through 1 µm filter (Pall, NP6P1001, Saint Germain en Laye, France) yielding the representative root extract of *Morus alba*. L. The reference extract of commercial *Morus alba* L. roots (Cortex Mori Albae Radicis/Sang Bai Pi, CapsulCN International, Ruian, China), used for collagenase inhibition studies, was prepared in the same manner.

### 4.8. UHPLC Analyses

All samples were analyzed using the UHPLC Shimadzu Nexera X2 system (Shimadzu, Kyoto, Japan) with a photodiode-array detector (applied range: 220–370 nm, detection at 265 nm) coupled to a mass spectrometer LCMS2020 (electrospray ionization in a negative ion mode, *m/z* 200–1000), using a Kinetex EVO C18 reverse phase column (150 mm × 2.1 mm, 2.6 µm, Phenomenex, Torrance, CA, USA), maintained at 40 °C during all analyses. The mobile phase was composed of water containing 0.1% vol. of formic acid (**A**) and pure acetonitrile (**B**), delivered at 0.5 mL/min with the gradient of B phase as follows: 5–72.5% (0–22 min); 72.5–90% (22–22.1 min); hold at 90% (22.1–23.9 min); 90–5% (23.9–24 min), hold at 5% (24–26 min).

### 4.9. Target Binding^TM^ Technology to Determine Collagenase Affinity

The patented Target Binding^TM^ approach is a method for determining the affinities between the ligands and protein target of interest. The method was described in the patent WO/2018/055053 (Salwinski, A. Method for determining the affinity between ligands and a target.

**Figure 8:**
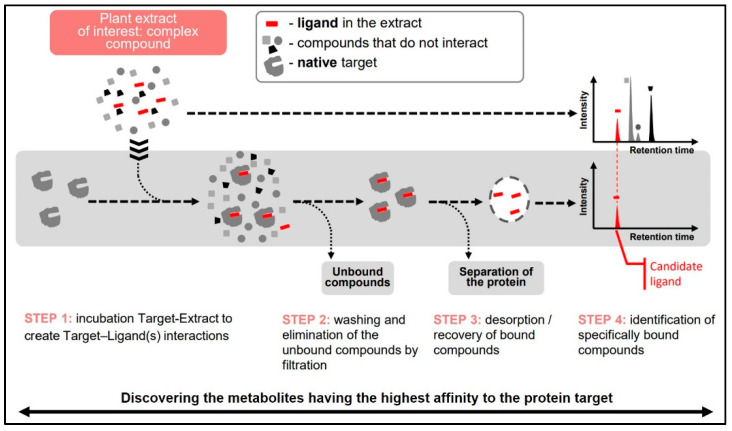
Different steps of the target binding strategy to identify ligand with an affinity to a protein target.

In the case of enzymes, it can be considered as a pre-screen test to find inhibitor candidates. The procedure of Target Binding^TM^ includes the following steps (Figure 8):

1. Binding: Incubation of the target protein with test mixture to form protein-ligand complexes;
2. Washing: Elimination of non-specifically bound compounds and separation of the target-ligand complexes from the incubation mixture;
3. Denaturation of target-ligand complexes to desorb bound ligands and elimination of denatured target by precipitation/centrifugation;
4. Analysis of the supernatant containing dissociated ligands and the initial test sample to determine Relative Affinities (RA) of individual components of the mixture.

In this assay, collagenase from *Clostridium histolyticum*, type IA (Sigma-Aldrich, ref. C9891, Saint-Quentin-Fallavier, France) was prepared at 5 mg/mL in a 50 mM ammonium acetate buffer solution. The enzymatic solution was added to the plant extract (20 mg of ground roots/mL of pure EtOH) and incubated at room temperature for 10 min, in order to ensure the binding of the ligands to the enzyme. This mixture was then filtered through a 10 kDa cut-off centrifugal filter by centrifugation at 14,000 g, until the solvent was eliminated. The enzyme-ligand complexes, deposited on the surface of the ultrafiltration membrane, were re-suspended in a 50 mM ammonium acetate buffer solution that was centrifuged at 14,000× *g* until the solvent was eliminated. The enzyme-ligand complexes were then re-suspended in water and added to acetonitrile, in order to denature and precipitate the protein and desorb the ligands. The supernatant was then analyzed using HPLC Shimadzu Nexera X2 (Shimadzu, Kyoto, Japan).

### 4.10. Collagenase Inhibition Activity

The inhibition test of collagenase used in this paper was developed in our laboratory using the classic synthetic substrate of this enzyme—FALGPA. The rate of enzymatic-driven FALGPA hydrolysis to FAL and GPA was followed by HPLC, instead of commonly applied microplate reader or UV-Vis spectrometer. This protocol was based on Van Wart′s work [21]. The principle of the assay is based on the catalytic hydrolysis of a synthetic substrate of collagenase-peptide FALGPA into GPA and FAL by the collagenase. The FALGPA conversion rate into FAL is proportional to the activity of enzyme and inversely proportional to the inhibitory properties of the test sample. Briefly, a volume of 100 µL of 1.5 mM solution of FALGPA (Bachem, ref. 4006713.0025, Bubendorf, Switzerland) in 50 mM tricine buffer with 10 mM calcium chloride and 400 mM sodium chloride, pH 7.5, was mixed with a volume of 10 µL of test sample or pure solvent of the test sample (positive control), followed by a volume of 10 µL of 0.05 mg/mL of *Clostridium histolyticum* collagenase (type IA, Sigma-Aldrich, ref. C9891, Saint-Quentin-Fallavier, France). To determine the rate of enzymatic conversion, the content of FAL was quantified at the beginning of the reaction and 30 min after its initialization by stopping the enzymatic conversion of FALGPA by EDTA. Briefly, to quench the enzymatic conversion, a volume of 50 µL of the test sample was mixed with a volume of 50 µL of EDTA (0.2 M in water). The content of FALin the samples was then quantified by UHPLC method optimized to separate FALGPA and FAL Briefly, the analysis was conducted using Kinetex Biphenyl reverse phase column (150 mm × 2.1 mm, 2.6 µm, Phenomenex, Torrance, CA, USA), maintained at 40°C. The mobile phase was composed of water containing 0.1% vol. of formic acid (A) and pure acetonitrile (B), delivered at 0.5 mL/min with the gradient of B phase as follows: 5–41% (0–9 min); 41–90% (9–9.05 min); hold at 90% (9.05–11.50 min); 90–5% (11.50–11.55 min), hold at 5% (11.55–14.50 min). Sample injection volume was 5 µL and FALGPA/FAL detection at 338 nm. Residual activity (ActR%) and the inhibition degree (Inh%) of collagenase in the presence of test sample X were then calculated using the Equations (1) and (2), respectively.

(1) $$\mathrm{Act}{R\%}_{\mathrm{EchX}}=\frac{{\mathrm{AUC}}_{\mathrm{EchX},T=30}^{\mathrm{FAL},\;338\mathrm{nm}}-{\mathrm{AUC}}_{\mathrm{EchX},T=0}^{\mathrm{FAL}\;338\mathrm{nm}}}{{\mathrm{AUC}}_{\mathrm{BL},T=30}^{\mathrm{FAL},\;338\mathrm{nm}}-{\mathrm{AUC}}_{\mathrm{BL},T=0}^{\mathrm{FAL},\;338\mathrm{nm}}}\;\times 100\%$$

(2) $$\mathrm{Inh}{\%}_{\mathrm{EchX}}=100\%-\mathrm{Act}{\%}_{\mathrm{EchX}}$$

where: ActR%—residual activity; Inh%—inhibition degree; AUC—peak area of FAL; EchX—tested sample; BL—blank.

### 4.11. Comparison of Collagenase Inhibition Potential of N- and the Commercial Extract of Morus alba L. Roots

The anti-collagenase activity of N- root extract and commercial root extract of *Morus alba* L. (50 mg of dry root mass/mL of 1,3-propanediol/water 80/20 *v/v*) were compared by evaluation of ActR% for pure extracts (yielding the final concentration of both extracts in the reaction mixture at 8.33%).

### 4.12. Evaluation of the Synergic Effect of Metabolites on the Inhibition of Collagenase Enzyme

Inhibitory properties of all major metabolites of *Morus alba* L. (moracenin A, moracenin B, kuwanon C, morusin) were evaluated at 20.8 µM, a concentration close to IC_50_ of moracenin B and compared with the inhibition potential of N- extract containing these markers at the total concentration of 20.8 µM. The total content of the markers in the raw extract N- (50 mg of dry powder/mL in 1,3-propanediol/water (80/20)) was 1519.1 µM. To yield the total content of the markers at 20.8 µM, the extract was tested at 1.37%.

### 4.13. Matrisome Related Genes Expression Study

Normal human foreskin dermal fibroblasts NHDFs were used for the experiments (ATCC, reference CRL-2522, LGC Promochem). N- extract at 0.2% was applied on fibroblasts for 24 h (in triplicate). The transforming growth factor-β1 at 20 ng/mL was used as a positive control. After treatment, total RNAs were extracted using the RNeasy Mini Kit (Qiagen ref. 74106,) from Qiagen (Hilden, Germany), according to manufacturer instructions. Then the extracted RNAs were quantified (Ultrospec 1100 Pro-Amersha, GE healthcare, Amersham, UK) and their integrity was analyzed through the use of capillary electrophoresis (Agilent Bioanalyzer 2100—Agilent RNA 6000Nano Kit, 5067-1511). For specific genes that target matrisome-related genes, gene expression levels were quantified by a qRT-PCR method and through the use of TaqMan^®^ cards from Applied Biosystems (Carlsbad, CA, USA). The following matrisome-related genes were studied: Col3A1 (assay ID -Hs00943809_m1), Cyr61-(assay ID Hs00998500_g1) and MMP-1 (assay ID Hs00899658_m1). The normalization method was based on the reference to a housekeeping gene, that is, glyceraldehyde-3-phosphate dehydrogenase (assay ID Hs02758991_g1). Data Assist Software v3.01 (Applied Biosystems, Foster City, CA, USA) was applied for data analysis, based on the comparative CT (ΔΔCT) method for calculating relative quantitation of gene expression. This was achieved through a combination of statistical analysis and interactive visualization. A two-sample, two-tailed Student′s *t*-test comparing the ΔCT values of the two groups is performed and a *p*-value was calculated.

### 4.14. Procollagen Type I C Propeptide (CICP) Quantification

Normal human dermal fibroblasts (NHDFs) were seeded in 24 well plate and cultivated in DMEM 1% fetal bovine serum (Gibco, ref. 10270-106, Thermofisher Scientific, Waltham, MA, USA), which was supplemented with penicillin/streptomycine (Gibco, ref. 15140-122, Thermofisher Scientific) for 72 h. Then, the culture medium was replaced by a fresh DMEM medium that contained MAE at 0.00625% and 0.0125% or the corresponding solvent (1.3 propanediol at 80%) at the same concentrations for 24 h before the first UVB irradiation session (125 mJ/cm^2^). The DMEM medium was renewed each day with Morus alba L. extract (MAE) or the solvent, before each UVB irradiation session. NHDFs were UVB irradiated once each day for a period of 4 days. After the last UVB irradiation session, cells were allowed to recover for a period of 72 h in serum-free DMEM medium. Then, at the end of this recovery period, the supernatants were collected for the dosage of procollagen type I C propeptide (CICP) and viability assay (MTS: 3-4,5-dimethylthiazol-2-yl-5-(3-carboxy-methoxyphenyl)-2-(4-sulfophenyl)-2*H* tetrazolium). CICP concentration was assessed by ELISA (enzyme linked immunosorbent assay), according to the manufacturer’s instructions (CICP microvue Quidel kit, ref. 8003, Tecomedical). The CICP concentrations measured were normalized to MTS viability data. The cell viability was determined through the use of an MTS (3-(4,5-dimethylthiazol-2-yl)-5-(3-carboxymethoxyphenyl)-2-(4-sulfophenyl)-2*H*-tetrazolium) assay (Promega, ref. G3581). 0.008% SDS (Sodium dodecyl sulfate) (VWR, ref. 444464T) was used as a reference molecule to validate the cytotoxicity MTS assay. The experiment was done in triplicate.

### 4.15. Clinical Investigation

This randomized double-blind placebo controlled clinical trial was conducted in Grenoble (France) by Dermatec. The study was conducted in accordance with the principle of the Declaration of Helsinki and the guidelines of the International Conference on Harmonization Good Clinical Practice, as applicable to a non-drug study. Twenty-two Caucasian females, who had given their written and informed consent, were enrolled in this clinical study. The study duration was 8 weeks with an intermediate time point at 4 weeks. The volunteers in the study had an age range of 45–70 years and were presenting wrinkles, as well as a dull and uneven complexion. Subjects applied the placebo and the product containing the active ingredient N- root extract twice a day at 1% on one half of their face. The formula used was the following—water, glycerin, glyceryl stearate (and) PEG-100 stearate, isopropyl myristate, cyclopentasiloxane, cetyl alcohol, cyclopentasiloxane (and) dimethiconol, N- root extract (1%), water (and) sodium hydroxide, phenoxyethanol, ethylhexylglycerin (and) tocopherol, chlorphenesin, carbomerv, perfume. The placebo formula did not contain N- root extract.

The parameters were measured through the use of a dermatoscope C-cube. This device was deployed to take high 2D and 3D resolution pictures on wrinkle depth (Sv) and skin smoothness (Sz). While Sv measures the valley depth, revealing the deepest wrinkles, Sz represents the total amplitude (difference between highest peak and deepest valley), correlated with skin smoothness. The plumping effect of the product was also determined through the use of an 11 point scoring scale. A 0 score means sagging non-firm skin, while a 10 score means a tonic skin, that is firm and plumped up. The plumping effect was visually assessed by a trained assessor.

## 5. Conclusions

The aeroponic-based Plant Milking technology has proven to be a very efficient technique to produce *M. alba* L. root extracts. This is both in terms of the novelty of the phytochemical profile of the extract itself and for the high concentration in prenylated flavonoids found in this extract.

Due to the performances observed on mulberry tree, we have no doubt that the Plant Milking technology can be efficiently deployed to produce root extracts from virtually any kind of higher plant species.

Moreover, we have demonstrated that this technology allows for the development of an efficient cosmetic active ingredient with anti-aging properties for the skin.

## Figures and Tables

**Table 1:** Relative affinities of the components of *Morus alba* L. root extract to collagenase from *Clostridium histolyticum*.

Relative Affinity (RA) for Collagenase				
Morusin	Kuwanon C	Moracenin B (reference)	Moracenin A	Wittiorumin F
0.9	0.9	1.0	1.1	1.1

**Table 2:** Evaluation of collagenase inhibitory activity of N- extract and its prenylated compounds in comparison to a commercial extract of *Morus alba* L. roots using UHPLC technique.

	Condition	ACtR% (Relative Collagenase Activity)	Inhibition Rate (%)
Collagenase inhibition synergy by the constituents of N-	Blank	100%	0%
	N- extract containing a 20.8 µM equivalent prenylated compounds (N-, 1.37%) (Moracenin B: 6 µM, Kuwanone C: 6.7 µM, Morusin: 4 µM, Moracenin A: 4.1 µM)	27%	73%
	Moracenin B (20.8µM)	58%	42%
	Kuwanon C (20.8µM)	62%	38%
	Morusin (20.8 µM)	68%	32%
	Moracenin A (20.8 µM)	38%	62%
Comparison with the commercial roots	N- extract, 8.33%	9%	91%
	Extract of commercial *Morus alba* L. roots (8.33%) prepared in the same way as N-	105%	0%

**Table 3:** Genes modulated in fibroblasts when treated for 24 h with N- at 0.2%. The relative expression (RQ) of each gene was calculated in comparison to fibroblasts treated with the vehicle dimethyl sulfoxide (DMSO) at 1%.

Name of the Gene Modulated	Relative Expression (RQ)	*p* Value
Cysteine rich angiogenic protein 61 (CCN1)	0.7	0.07
Matrix metalloproteinase 1 (MMP1)	0.6	0.0066
Collagen 3 aplha 1 subunit (COL3A1)	1.49	0.01

**Figure A3:**
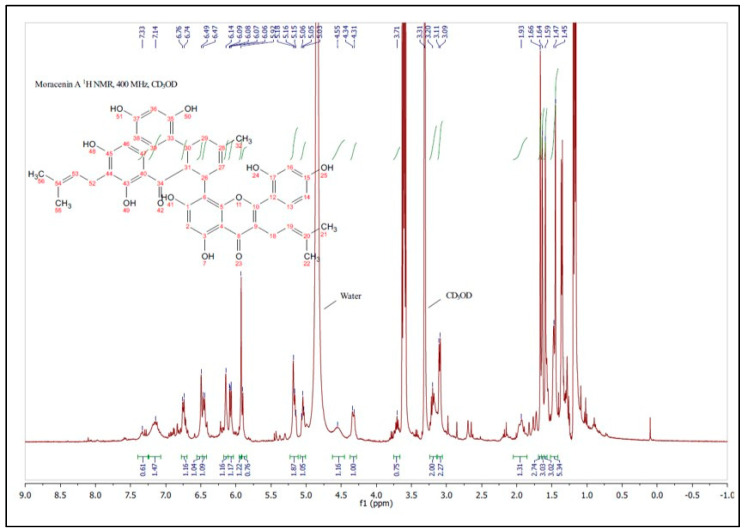
^1^H-NMR (400 MHz, CD_3_OD) spectrum of the compound Moracenin A.

## Appendix B

### Plant Metabolite Identification

The metabolites of *M. alba* L. root extracts were identified using mass spectrometry (in negative ion mode) based on the detection of pseudo-molecular ions [M − H]^−^. After purification from the *Morus alba L.* extract the molecular structures were confirmed by NMR) analyses.

The NMR spectra of the molecules identified (moracenin B, moracenin A, kuwanon C and morusin) are presented in Figure A2, Figure A3, Figure A4, Figure A5, Figure A6, Figure A7 and Figure A8 below. The chemical structures of the molecules are presented in Figure A9.
